# Loss of Projections, Functional Compensation, and Residual Deficits in the Mammalian Vestibulospinal System of *Hoxb1*-Deficient Mice^1,2,3^

**DOI:** 10.1523/ENEURO.0096-15.2015

**Published:** 2015-12-26

**Authors:** Maria Di Bonito, Jean-Luc Boulland, Wojciech Krezel, Eya Setti, Michèle Studer, Joel C. Glover

**Affiliations:** 1 Institute of Biology Valrose, UMR 7277, University of Nice Sophia Antipolis, 06108 Nice, France; 2 Institute of Biology Valrose, INSERM, U1091, 06108 Nice, France; 3 Institute of Biology Valrose, CNRS, UMR 7277, 06108 Nice, France; 4 Division of Physiology, Department of Molecular Medicine, University of Oslo, 0317 Oslo, Norway; 5 Norwegian Center for Stem Cell Research, Oslo University Hospital, 0317 Oslo, Norway; 6 Institut Génétique Biologie Moléculaire Cellulaire, CNRS UMR 7104, INSERM U 964, 67404 Illkirch Cedex, Strasbourg, France

**Keywords:** brain patterning, functional compensation, *Hox* genes, reticulospinal, vestibulo-ocular, vestibulospinal

## Abstract

The genetic mechanisms underlying the developmental and functional specification of brainstem projection neurons are poorly understood. Here, we use transgenic mouse tools to investigate the role of the gene *Hoxb1* in the developmental patterning of vestibular projection neurons, with particular focus on the lateral vestibulospinal tract (LVST). The LVST is the principal pathway that conveys vestibular information to limb-related spinal motor circuits and arose early during vertebrate evolution. We show that the segmental hindbrain expression domain uniquely defined by the rhombomere 4 (r4) *Hoxb1* enhancer is the origin of essentially all LVST neurons, but also gives rise to subpopulations of contralateral medial vestibulospinal tract (cMVST) neurons, vestibulo-ocular neurons, and reticulospinal (RS) neurons. In newborn mice homozygous for a *Hoxb1*-null mutation, the r4-derived LVST and cMVST subpopulations fail to form and the r4-derived RS neurons are depleted. Several general motor skills appear unimpaired, but hindlimb vestibulospinal reflexes, which are mediated by the LVST, are greatly reduced. This functional deficit recovers, however, during the second postnatal week, indicating a substantial compensation for the missing LVST. Despite the compensatory plasticity in balance, adult *Hoxb1*-null mice exhibit other behavioral deficits that manifest particularly in proprioception and interlimb coordination during locomotor tasks. Our results provide a comprehensive account of the developmental role of *Hoxb1* in patterning the vestibular system and evidence for a remarkable developmental plasticity in the descending control of reflex limb movements. They also suggest an involvement of the lateral vestibulospinal tract in proprioception and in ensuring limb alternation generated by locomotor circuitry.

## Significance Statement

The mammalian motor system is constructed from neuron groups that acquire specific functional identities in part through the action of patterning genes such as those in the *Hox* gene family. Here, we assess the role of the *Hoxb1* gene in the development of the murine vestibular system. Hoxb1 function is required to generate specific groups of vestibular neurons, in particular neurons that give rise to the lateral vestibulospinal tract (LVST). The lack of the LVST resulting from the absence of Hoxb1 function leads to an initial deficit in vestibulospinal reflexes, but these recover over the course of several days, indicating a pronounced functional compensation. Subtle behavioral deficits are maintained into adulthood, suggesting additional roles for the LVST in motor control, most notably in proprioception and interlimb coordination during locomotion.

## Introduction

The vestibular system appeared early during vertebrate brain evolution, and connections from the vestibular nuclei to motoneurons in the brainstem and spinal cord are highly conserved within the vertebrate radiation (Díaz and Glover, 2002; Beisel et al., 2005; Duncan and Fritzsch, 2012; Straka et al., 2014). Vestibular projections also appear early during brain development and are patterned by highly stereotyped blueprints of gene expression (Glover and Petursdottir, 1991; Díaz et al., 1998; Glover, 2000, 2003; Pasqualetti et al., 2007; Straka and Baker, 2013). Despite its conserved and stereotyped connectivity, the vestibular system exhibits marked adaptive plasticity in the face of sensorimotor mismatch, activity imbalances, or outright damage (du Lac et al., 1995; Minor, 1998; Quinn, 1998; Darlington and Smith, 2000; Raymond and Lisberger, 2000; Yates et al., 2003; Gliddon et al., 2005; Gittis and du Lac, 2006; Ronca et al., 2008; Cullen et al., 2009; Horak, 2009; Dutia, 2010; McElvain et al., 2010; Jamon, 2014; Shin et al., 2014). The contribution of the vestibular system to balance is also affected negatively by aging, resulting in vertigo, discomfort, and falls in the elderly, which are associated with a high degree of morbidity and mortality (Ishiyama, 2009; Agrawal et al., 2013; Iwasaki and Yamasoba, 2014).

Principal vestibular descending projections include the separate ipsilateral medial vestibulospinal tract (iMVST) and contralateral medial vestibulospinal tract (cMVST), and the strictly ipsilateral lateral vestibulospinal tract (LVST; Glover and Petursdottir, 1988; Díaz et al., 2003; Pasqualetti et al., 2007). Of these, only the LVST projects along the entire spinal cord, and it is pivotal in regulating the activity of trunk and limb musculature to counteract perturbations of body position (Shinoda et al., 1988; Kuze et al., 1999; Rose et al., 1999; Boyle, 2000; Bácskai et al., 2002; Matesz et al., 2002; Kasumacic et al., 2010). A principal function is the activation of limb extensors and deactivation of limb flexors, asymmetrically around the body axis, to generate limb movements that maintain an upright body position when balance is lost (Wilson and Yoshida, 1969; Pompeiano, 1972).

Despite recent advances (Díaz et al., 1998; Pasqualetti et al., 2007; Kasumacic et al., 2010, 2015), we still lack a comprehensive understanding of how the LVST group arises developmentally, becomes specified to project along the LVST pathway, and selectively innervates different populations of spinal neurons. Indeed, we have very little information about the specific spinal targets of the LVST and therefore about how the LVST exerts its effects.

One way to better understand the development and function of the LVST is to use molecular genetic approaches to interrogate LVST neurons about their origins, connections, and physiological effects. The LVST group has been shown to derive predominantly from rhombomere 4 (r4) in both chicken and mouse (Díaz et al., 1998; Pasqualetti et al., 2007). Because the gene *Hoxb1* is instrumental in establishing the identity of r4, Chen et al. (2012) used a *Hoxb1* reporter mouse and a *Hoxb1*-null mouse to test the r4 origin of vestibulospinal neurons. Their results supported this origin and showed that inactivation of *Hoxb1* led to a loss of vestibulospinal neurons. However, they did not assess whether this manipulation was specific to the LVST group. This is important because the cMVST group also has an r4-derived component, as may other vestibular and bulbospinal projection neuron groups (Auclair et al., 1999). Nor did Chen et al. (2012) investigate the functional effects that the loss of r4-derived vestibulospinal neurons elicited.

**Figure 1.**
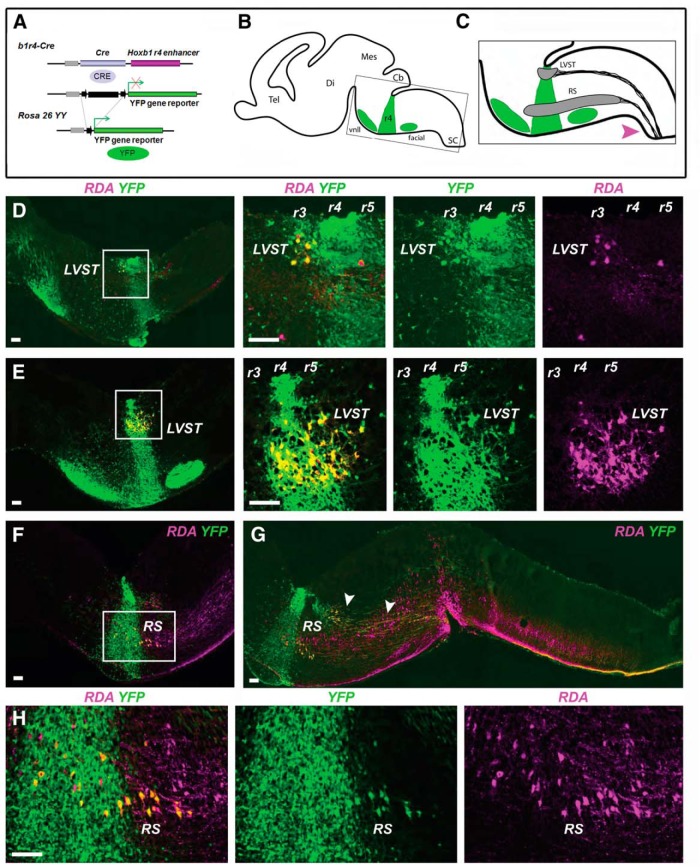
Fate mapping of LVST neurons and reticulospinal neurons. ***A***, The *b1r4-Cre* line, which expresses the *Cre recombinase* under the control of the *Hoxb1* r4 enhancer, is crossed with the *ROSA26YY* reporter line to label r4 derivatives by YFP expression. ***B***, ***C***, Schematic diagrams of E16.5 brain in the parasagittal plane illustrating the location of YFP-positive r4 and r4 derivatives (green), and the domains of the LVST group and the main population of ipsilaterally projecting reticulospinal (RS) neurons (gray). ***C***, Magenta arrowhead indicates the tracer application site used to retrogradely label vestibulospinal and reticulospinal neurons. ***D***, ***E***, All LVST neurons, including those in r3 and r5, derive from r4. The first panel in each horizontal series is a low-magnification image, with a white square indicating the region shown in the subsequent images. ***F–H***, A population of ipsilaterally projecting RS neurons in r4 and r5 derives from r4. ***G***, Arrowheads indicate LVST axons projecting just dorsal to the RS neurons. RDA/BDA labeling is depicted by magenta, YFP immunolabeling is depicted by green, and double labeling appears as varying hues of yellow and orange. Note that in this and subsequent figures some double-labeled neurons appear to have a magenta nucleus with surrounding yellow to orange cytoplasm; this is because the RDA/BDA can partition into the nucleus where the YFP is weak. Tel, Telencephalon; Di, diencephalon; Mes, mesencephalon; Cb, cerebellum; SC, spinal cord; vnll, ventral nucleus of lateral lemniscus; facial, VIIth cranial (facial) nerve motor nucleus. Scale bars, 200 µm.

Here, we use a transgenic mouse that expresses *Cre recombinase* under the control of the r4-specific enhancer element of *Hoxb1* (*b1r4-Cre* mouse; Di Bonito et al., 2013a; Fig. 1*A*) to make a comprehensive characterization of the contribution of r4 to the LVST, cMVST, iMVST, and nearby vestibulo-ocular (VO), vestibular efferent, and reticulospinal neurons. We then test the dependence of these neuron groups on Hoxb1 protein function using a constitutive *Hoxb1*-null mutant mouse, and investigate the resulting behavioral effects. We provide novel evidence about the origins of these projection and efferent neurons, and their dependence on *Hoxb1* expression for normal development. Our results also shed light on the role of the LVST and the capacity for functional reorganization when this important component of descending motor control is developmentally compromised.

## Materials and Methods

### Animals

In the *b1r4-Cre* transgenic line (Di Bonito et al., 2013a), the *Cre recombinase* gene is expressed exclusively in rhombomere 4 under the control of the *Hoxb1* r4 enhancer (Studer et al., 1994). The *b1r4-Cre* line was crossed with the *ROSA26YY* reporter line (Srinivas et al., 2001). In the double-heterozygous *b1r4-Cre/YFP* mice, r4 and r4 derivatives are selectively labeled by yellow fluorescent protein (YFP) expression. Wild-type mice generated from this crossbreeding were used as controls. The *Hoxb1*-null line (Di Bonito et al., 2013a) was crossed with the *b1r4-Cre/YFP* line to label r4 in the mutant background. A total of 61 mice were used in this study, including 11 *b1r4-Cre/YFP* embryos, 17 wild-type postnatal mice, and 12 embryos and 21 postnatal mice from the *Hoxb1*-null × *b1r4-Cre/YFP* cross.

All mice were raised on a B6D2 genetic background and housed in groups of two to four animals/cage in a 12 h (7:00 A.M. to 7:00 P.M.) light/dark cycle with food and water freely available. Pregnancies were identified and timed by the presence of vaginal plugs. All efforts were made to minimize the number of animals used and their suffering, in accordance with the European Communities Council directive 2010/63/EU for the care and use of animals. All procedures were conducted according to French ethical regulations, and this project received the approval from the local ethics committee (CIEPAL NCE/2014-209).

### Retrograde tracing with fluorescent dextran-amines

Pregnant dams were killed by cervical dislocation, subjected to a caesarean section, and embryonic day 16.5 (E16.5) embryos with decidua were removed and submerged in ice-cold (4°C), oxygenated (95% O2 and 5% CO2), artificial CSF [ACSF; containing the following (in mm): NaCl 128, KCl 3, d-glucose 11 CaCl2 2.5, MgSO4 1, NaH2PO4 1.2, HEPES 5 and NaHCO3 25]. Embryos were then decapitated at low cervical levels, and the brainstem was carefully dissected out. To maximize oxygenation, the cerebellum was removed, and the ACSF was exchanged every 10 min during the dissection.

To label vestibulospinal and reticulospinal neurons, we used the approach previously described by Glover (1995) and Kasumacic et al (2010). The spinal white matter at the level of the first cervical (C1) ventral root was cut unilaterally. The cut spanned the entire extent of the ventral and ventrolateral funiculi. Premade crystals of 3 kDa tetramethylrhodamine-conjugated dextran amines (RDA; catalog #D-3308, Invitrogen), alone or in combination (1:1 ratio) with biotin-conjugated dextran amine (BDA; catalog #D-7135, Invitrogen) were inserted into the cut. Four to six crystals inserted over a period of ∼3 min ensured continuous exposure of the cut axons to high tracer concentration. Preparations were then incubated in the dark for a period of ∼8 h to allow retrograde transport of the tracers to both ipsilaterally and contralaterally projecting vestibulospinal and reticulospinal neurons.

To label vestibulo-ocular neurons, the same procedure was performed after making a unilateral cut in the medial longitudinal fasciculus (MLF), approaching from the floor of the fourth ventricle, at the level of the pons/mesencephalon border, as described previously (Pasqualetti et al., 2007).

### Retrograding tracing with lipophilic dye

Whole heads of P8 *Hoxb1*-null and control mice (*n* = 3 for each genotype) were fixed overnight in 4% paraformaldehyde (PFA) in PBS. The heads were dissected ventrally to expose the inner ear. Vestibular efferent neurons were retrogradely labeled by placing a crystal of the fluorescent carbocyanine dye DiI (Molecular Probes catalog #D-282, ThermoFisher Scientific) unilaterally into the inner ear, ensuring contact with the peripheral nerve branches. To allow the DiI to diffuse, preparations were incubated for 3 months in PBS containing 0.025% sodium azide; the first two months at 37°C and thereafter at room temperature to minimize tissue degradation. The brain was then dissected free and vibratome-sectioned at 100 µm in the coronal plane, and the sections were mounted on slides for inspection under epifluorescence optics.

### Histology and microscopy

YFP^+^ neurons and axons were visualized both by the intrinsic fluorescence of YFP and amplification using an antibody that recognizes both YFP and GFP (anti-GFP; Molecular Probes catalog #A11122, ThermoFisher Scientific). Preparations were immersion fixed in 4% PFA in PBS, cryoprotected, frozen, and cryostat sectioned at 12 µm in the sagittal plane. Sections were mounted on glass slides and incubated in the primary anti-GFP antibody at 1:500 overnight at 4°C. They were then rinsed and incubated for 1 h at room temperature with either Alexa Fluor 488 (green)-conjugated goat anti-rabbit secondary antibody (Life Technologies catalog #A11034, ThermoFisher Scientific) and Alexa Fluor 594 (red)-conjugated streptavidin (Invitrogen Molecular Probes catalog #S32356, ThermoFisher Scientific), to amplify the RDA/BDA labeling, or with a biotinylated secondary anti-rabbit antibody (Life Technologies catalog #32054, ThermoFisher Scientific). The slides incubated with fluorescent secondary antibodies were then rinsed and coverslipped for inspection under epifluorescence optics. The slides incubated with biotinylated secondary antibody were rinsed and incubated with biotinylated horseradish peroxidase (HRP) preincubated with avidin, and rinsed and carried through the HRP reaction using DAB as the substrate, as previously described (Di Bonito et al., 2013a), before being coverslipped for inspection under conventional optics.

*In situ* hybridization using a probe for mRNA of the transcription factor GATA3 was performed as previously described (Di Bonito et al., 2013a).

Digital photographic images were obtained at 5× magnification using a Leica DM 6000B microscope equipped with Leica DFC310 FX color camera and processed in Adobe Photoshop CS5 software using the Photomerge function to obtain a panorama of each brain section. In all images obtained with this system, RDA/BDA labeling was red, YFP labeling was green, and double labeling appeared as varying shades of yellow to orange. In many cases, conjugated dextrans entered the nucleus where the YFP was weaker than in the cytoplasm, so that some double-labeled neurons appeared to have a red nucleus with surrounding yellow to orange cytoplasm. To assist the color blind, images were then additionally processed to replace red pixels with magenta pixels. Double labeling thus remained as yellow to orange. To assess double labeling of individual neurons, confocal *z*-stacks were obtained by laser scanning confocal microscopy at 40× magnification using a Zeiss LSM 710 confocal. Neuronal cell bodies were examined individually in the *x*-, *y*-, and *z*-planes by ZEN software. False colors were generated in all images to make RDA/BDA labeling magenta and YFP labeling green, and double labeling thus appeared as a pale whitish hue, in contrast to the yellow to orange hues that were obtained with the Leica DFC310 FX color camera system.

### Behavioral testing

#### Early postnatal period

Neonates and early postnatal mice have immature motor skills, particularly because they are not capable of bearing their own weight until the second postnatal week. For this reason, most adult behavioral tests cannot be used on neonates and young mice without substantial modification. We therefore developed specialized procedures and apparatus, and used these to test gender-balanced cohorts of *Hoxb1*-null mice (*n* = 21) and wild-type control mice (*n* = 14) from postnatal day 5 (P5) to P11. All tests were performed by an investigator blinded to the genotype of the mouse.

##### Open field swimming test.

To assess general locomotor ability, P5 mice were placed in a water-filled rectangular pool (210 × 165 mm) and allowed to swim freely while being filmed on video from above at 25 Hz with a Nikon Micro-Nikkor 55 mm 1:3.5 objective. To avoid exhaustion and hypothermia, the swimming time was limited to 20 s, and the water temperature was maintained at ∼36°C. Tracking for each mouse was performed in ImageJ (Rasband, 1997) using the *Manual Tracking* plugin (Cordeliéres, 2006). Analyses of trajectory, time spent in the center or the periphery of the pool, number of rotations, and minimum/maximum/average speeds were performed using an in-house program coded in LibreOffice Basic (unpublished).

##### Vestibulospinal reflex test.

To test the vestibulospinal reflex in P5–P11 mice, we engineered a small apparatus designed to rotate a mouse around its longitudinal axis. In wild-type mice, this rotation produces a compensatory extension of the hindlimb on the side toward which the mouse is turned, indicating an attempt to recover balance. The apparatus was composed of three different parts. The first was a Teflon mouse holder (available in multiple sizes to fit mice of different ages). The holder retains the mouse in a dorsoventral grip, leaving the limbs free. The holder was affixed to the rotating part of the device at a right angle so that the longitudinal axis of the mouse was aligned with the rotation axis. The rotation was generated by a spiral torsion spring. A stopper was used to maintain the spring in tension so that the removal of the stopper was followed immediately by rotation, which was limited to 90° by a second stopper. Thus, all mice were subjected to a standardized rotation axis (roll axis), speed (1500°/s = 26.2 rad/s), and angle (90°). The rotating part was in turn affixed to a stand of a height that aligned the rotation axis with the center of the video camera objective (Micro-Nikkor 55 mm 1:3.5, Nikon). Rotation of the mice and compensatory movements of the limbs were recorded at 200 frames/s (Integrated Design Tools, Inc.). Hindlimb extensions were scored visually as a clear change from an initial flexed position (both hindlimbs had to be flexed initially for the trial to be included) to an obviously extended position.

#### Adults

All tests were performed on gender-balanced cohorts of *Hoxb1*-null mice (*n* = 10) and wild-type control mice (*n* = 7). Most mice were tested at 13-16 weeks of age, except for two mice in each group, which were tested at 28 weeks of age. Because the performance of each older mouse did not differ from the rest of the group with corresponding genotype across all tests, they were included in the analyses, and results from mice at the two ages were pooled. All mice were tested in the following battery of behavioral tests in the order described below, over a period of 4 consecutive days.

##### Open field test.

Locomotor activity was tested simultaneously in two open field arenas (40 × 40 × 40 cm) made of Plexiglas and with white floors. The arenas were placed in a room homogeneously illuminated at ∼150 lux. Each mouse was placed on the periphery of the arena and allowed to explore the arena freely for 30 min, with the experimenter hidden from the sight of the animal. Animal behavior was recorded with a video camera, and analyses were performed *post hoc* using EthoVisionXT software (Noldus). Behavioral parameters were calculated automatically with the exception of unsupported rearing (rearing on hindlimbs without support against the wall of the arena) and leaning (rearing on hindlimbs with support against the wall of the arena), which had to be identified by the investigator.

##### Notched beam test.

Here we used a modified version of the notched beam paradigm, which was originally conceived to test motor coordination of the hindlimbs (Duchon et al 2011; Waanders and Lipp, 1989). Briefly, locomotion was assessed on a 1-m-long, 17-mm-wide wooden beam with a flat upper surface engraved with regular, square, 17-mm-deep, 17-mm-wide, and 17-mm-long incisions spaced every 17 mm, with a box providing a secure hiding place at one end. During the habituation phase, each mouse was first placed in the box for 30 s. It was then removed from the box and placed twice on the beam at increasing distances from the box, and was allowed to traverse the beam to reach the box and hide in it for 30 s. During the test, the mouse was placed at the end of the beam opposite the box, and the time to reach the box, the number of paw slips during the traverse, and the number of hops (defined as synchronous movements of the two hindlimbs from one square to another) during the traverse were scored. The test was repeated three times at 30-60 s intervals.

##### Contact righting reflex.

The test was performed as described by Mandillo et al. (2008). Briefly, mice were placed in a narrow, 3-cm-wide, clear Plexiglas cylinder and allowed to come to rest. The cylinder was then held in a horizontal position and was rotated rapidly by 180° to bring the mouse to a supine position. The time required for the mouse to return to its original prone position was then measured.

##### Linear swimming test.

The test was performed in a 1-m-long Plexiglas tank (6 cm wide × 30 cm high) filled with water (20 cm deep) at a temperature of 21-23°C, as described by Romand et al. (2013). Briefly, for habituation, animals were placed for 30 s on a platform at one of the extremities of the tank, and subsequently they were gently lowered into the water on the opposite end of the tank. The time required to reach the platform was measured, and the body position and types of movements made during swimming were filmed on video and analyzed.

### Statistics

Differences of means were tested using the Student’s *t* test or the Mann–Whitney *U* test, or using ANOVA on repeated measures (RMANOVA) when the evolution of performance within a group was analyzed over the course of test epochs. *Post hoc* statistical comparisons of behavioral performance in *Hoxb1*-null and control mice were performed using the protected least significant difference (PLSD) Fisher test, as indicated in the corresponding figures.

## Results

### Developmental origins of vestibular projection neurons and reticulospinal neurons

Previous fate-mapping studies in chicken and mouse embryos have assessed the rhombomeric origins of vestibular projection neurons through fate mapping using quail-chicken chimeras and transgenic mice, respectively (Díaz et al., 1998; Pasqualetti et al., 2007; Chen et al., 2012). However, in the mouse, putative r4 origins have been assessed only indirectly using either a *r3-r5 lacZ* reporter mouse (Pasqualetti et al., 2007) or a *Hoxb1-GFP* reporter mouse in which the GFP is initially expressed from r4 into the spinal cord and only later restricted to r4 (Chen et al., 2012). Furthermore, in neither the chicken embryo nor these two mouse lines were the origins of specific neuron subpopulations residing in r4 determined unambiguously, since these studies did not categorically identify neurons that were double labeled by retrograde tracing and reporter gene expression or neurons devoid of reporter gene expression within r4. Here, by using *b1r4-Cre*/*YFP* mice, in which YFP staining is restricted to r4 from the onset of expression (Fig. 1*A*; Di Bonito et al., 2013a), combined with RDA/BDA retrograde labeling, we have been able to provide an accurate and comprehensive assessment of the contribution of r4 to vestibular projection neurons and reticulospinal neurons. We retrogradely labeled vestibulospinal and reticulospinal neurons (*n* = 5 preparations) or vestibulo-ocular neurons (*n* = 3 preparations) unilaterally to distinguish the different groups that project ipsilaterally and contralaterally.

#### LVST neuron group

Using the *b1r4-Cre* mouse line (Fig. 1*A–C*), we could show that essentially all LVST neurons originated from r4, including those located in r3 and r5 that migrate there from r4 prior to E16.5 (Fig. 1*D*,*E*). RDA/BDA-labeled, YFP^+^ axons could be followed from the LVST neuron group toward the spinal cord along the trajectory of the LVST (Fig. 1*C*,*G*, arrowheads). Because retrograde labeling is not 100% efficient, we cannot rule out that a few LVST neurons do not originate from r4, but the overwhelming majority clearly does.

#### Reticulospinal neurons

Reticulospinal neurons are located in and presumably derive from multiple rhombomeres in the mouse, including r4 (Fig. 1*C*; Auclair et al., 1999). However, rhombomere-specific fate mapping of the reticulospinal neurons has not been performed in rodents. Here, we could determine that a subpopulation of ipsilaterally projecting reticulospinal neurons in r4 and r5 derives from r4 (Fig. 1*F–H*). Thus, at least some reticulospinal neurons acquire their locations through inter-rhombomeric migration.

#### iMVST and cMVST neuron groups

**Figure 2.**
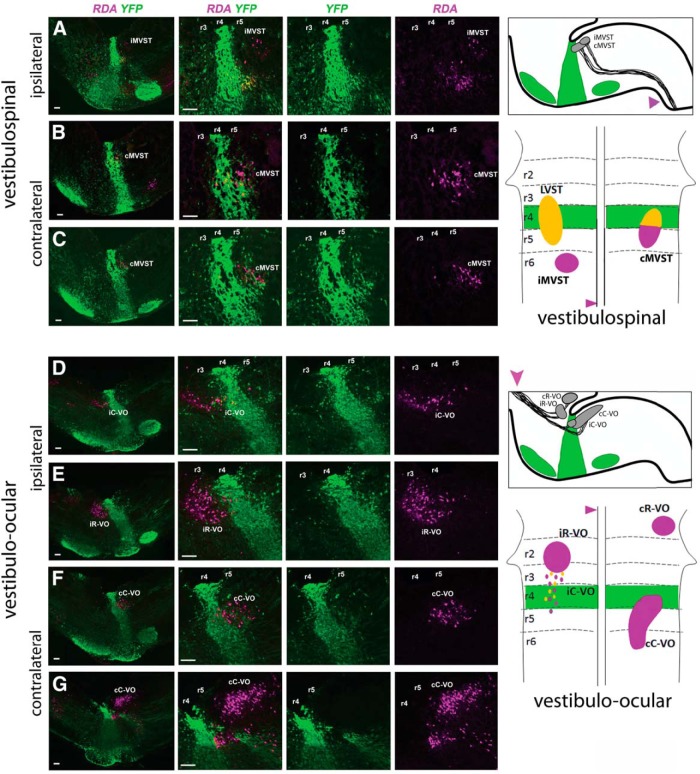
Fate mapping of other vestibulospinal neurons and of vestibulo-ocular neurons. ***A***, No iMVST neurons derive from r4. ***B***, ***C***, cMVST neurons located within r4 derive from r4, whereas those located in r5 do not. The first image in each horizontal sequence is at low magnification, and the subsequent images show higher magnification. RDA/BDA labeling is depicted by magenta, YFP immunolabeling is depicted by green, and double labeling appears as varying hues of yellow and orange. To the right are shown two schematic diagrams. The top one shows the location of YFP^+^ r4 and r4 derivatives (green), and the domains of the iMVST and cMVST groups (gray) in a parasagittal view of the brainstem. The magenta arrowhead indicates the site of tracer application used to retrogradely label vestibulospinal neurons. The lower one shows a dorsal view of the brainstem, indicating the rhombomeric domain (r4 in green), and the locations and rhombomeric origins of the three vestibulospinal groups. Magenta indicates a non-r4 origin and yellow (double-labeled YFP and RDA) indicates an r4 origin. ***D***, ***E***, Residual iC-VO neurons in r4 and some scattered ipsi VO neurons in r3 derive from r4 (***D***), but no iR-VO neurons derive from r4 (***E***). ***F***, ***G***, No cC-VO neurons derive from r4, although a subpopulation migrates into r4 (***F***). To the right are shown two schematic diagrams. The top one shows the location of YFP^+^ r4 and r4 derivatives (green), and the domains of the vestibulo-ocular groups (gray) in a parasagittal view of the brainstem. The magenta arrowhead indicates the site of tracer application used to retrogradely label vestibulo-ocular neurons. The bottom one shows a dorsal view of the brainstem, as above, indicating the rhombomeric origins and eventual locations of the vestibulo-ocular groups. Magenta indicates a non-r4 origin, and yellow (double-labeled YFP and RDA) indicates an r4 origin. Scale bars, 200 µm.

None of the iMVST neurons derives from r4, consistent with their origin from r6 (Fig. 2*A*; Díaz et al., 1998; Pasqualetti et al., 2007). The cMVST neurons that reside in r4 clearly originated from r4 (Fig. 2*B*), whereas the cMVST neurons in r5 clearly did not (Fig. 2*C*). Thus, the location of these two cMVST subpopulations in adjacent rhombomeres reflects primarily differential rhombomeric origins and not migration from a single rhombomere, as is the case for the LVST neurons.

#### Vestibulo-ocular neuron groups

Four different VO neuron groups project to the trochlear and oculomotor nuclei in both the chicken and the mouse, and these derive from regions either rostral [ipsilateral rostral-VO (iR-VO) group, contralateral rostral-VO (cR-VO) group] or caudal [ipsilateral caudal-VO (iC-VO) group, contralateral caudal (cC-VO) group] to r3 (in the mouse; Pasqualetti et al., 2007) or r4 (in the chicken; Díaz et al., 1998). In addition, there are scattered ipsilaterally projecting VO neurons lying in r3 (mouse) or r4 (chicken) between the iR-VO and iC-VO groups. Because it has not been determined whether these scattered neurons belong to the iR-VO or the iC-VO group, or whether they represent a separate, sparse group, we refer to them simply as “scattered ipsi VO neurons.” In the mouse, the iC-VO group is noteworthy because after first appearing (by E11.5), it dwindles markedly in number with subsequent development (through as yet undetermined mechanisms) such that by E16.5 only scattered iC-VO neurons remain in r4 and r5 (Pasqualetti et al 2007). Thus, the VO projection neurons in the E16.5 mouse present as three distinct groups, the cR-VO, cC-VO, and iR-VO groups, along with a “tail” of ipsilaterally projecting VO neurons that comprises the scattered ipsi VO neurons in r3 and the residual iC-VO neurons in r4 and r5.

Here, we could show that some of the scattered ipsi VO neurons in r3 and some of the residual iC-VO neurons in r4 originated from r4 (Fig. 2*D*). We also determined that no cR-VO (data not shown), iR-VO (Fig. 2*E*), or cC-VO neurons (Fig. 2*F*,*G*) originated from r4, although some cC-VO neurons migrate into r4 (Fig. 2*F*). Thus, although minor inter-rhombomeric migration to or from r4 occurs, this contributes relatively little to the patterning of the VO projection neuron groups.

### Loss of the LVST group, the r4-derived portion of the cMVST group, and some r4-derived reticulospinal neurons in *Hoxb1*-null mice

**Figure 3.**
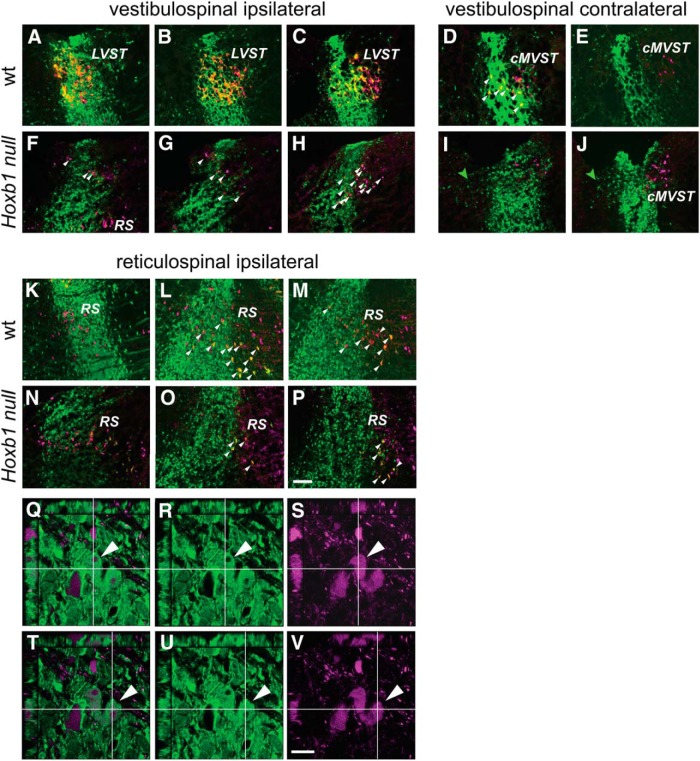
Loss of vestibulospinal neurons and depletion of reticulospinal neurons in the *Hoxb1*-null mutant. ***A–J***, Loss of vestibulospinal neurons. Comparison of the two vestibulospinal groups that derive wholly or partly from r4 in the wild-type (LVST, ***A–C***; cMVST, ***D***, ***E***) and the *Hoxb1*-null mutant (***F–J***) shows a complete absence of r4-derived LVST and cMVST neurons. The white arrowheads in ***D*** indicate examples of r4-derived cMVST neurons. Those in ***F–H*** indicate examples of non-r4-derived spinally projecting neurons in the *Hoxb1*-null mutant in the area where the LVST is normally located. The green arrowheads in ***I*** and ***J*** indicate non-spinally projecting r4-derived cells that migrate into r3 within the vestibular nuclear complex in the *Hoxb1*-null mutant. ***K–P***, Depletion of r4-derived reticulospinal neurons. Reticulospinal neurons derived from r4 (white arrowheads) are more numerous in the wild-type (***K–M***) than in the *Hoxb1*-null mutant (***N–P***). RDA/BDA labeling is depicted by magenta, YFP immunolabeling is depicted by green, and double labeling appears as varying hues of yellow and orange. ***Q–V***, Examples of confocal *z*-stacks to demonstrate double labeling, in this case of two reticulospinal neurons. Each panel shows a *z*-stack viewed in the *x–y* plane and from the *x–z* and *y–z* faces, with *x* and *y* transects intersecting at a reticulospinal neuron that is double labeled (one in ***Q–S***, another in ***T–V***). In each row of panels, the right panel shows only RDA (magenta), the middle panel shows only YFP (green), and the left panel shows a merge of the two (note that here the magenta and green combine to create a pale white, as opposed to the yellow/orange that depicts double labeling in the panels above; see Materials and Methods). RS, Reticulospinal neurons. Scale bars: ***A–P***, 200 µm; ***Q–V***, 20 µm.

Next, we used retrograde labeling in the *Hoxb1*-null mouse (labeling vestibulospinal and reticulospinal neurons unilaterally, *n* = 3; labeling vestibulo-ocular neurons unilaterally, *n* = 3) to test the dependence of r4-derived neuron populations on *Hoxb1* expression. First, we demonstrated that the LVST neuron group was severely depleted if not absent in E16.5 *Hoxb1*-null embryos (Fig. 3*F–H*). This is in accordance with the finding by Chen et al. (2012) that vestibulospinal neurons in the lateral vestibulospinal nucleus are lost in a different *Hoxb1*-null mouse strain. No retrogradely labelled YFP^+^ neurons were found in r3, r4, or r5 in the region that normally contains the LVST group (Fig. 3*A–C*,*F–H*), and there was no sign of YFP^+^ axons along the trajectory of the LVST. A few retrogradely labeled YFP-negative neurons were present in the area normally occupied by the LVST (Fig. 3*F–H*, arrowheads). These, however, numbered only a few tens of neurons (Fig. 3*F–H*, example where they are particularly numerous), whereas the LVST group normally contains many hundreds and perhaps well over a thousand neurons. The identity of these non-r4-derived neurons remains unclear.

The r4-derived portion of the cMVST group was also missing, leaving only the r5-derived portion (Fig. 3*D*,*E*,*I*,*J*). Thus, all r4-derived vestibulospinal neurons were either lost or transformed to another phenotype in the absence of Hoxb1 function, as previously shown for other r4-derived structures (Di Bonito et al., 2013a,b). As expected, the iMVST group (data not shown), as well as the r5-derived portion of the cMVST group, neither of which originate from r4, were unaffected in the *Hoxb1*-null mice.

**Figure 4.**
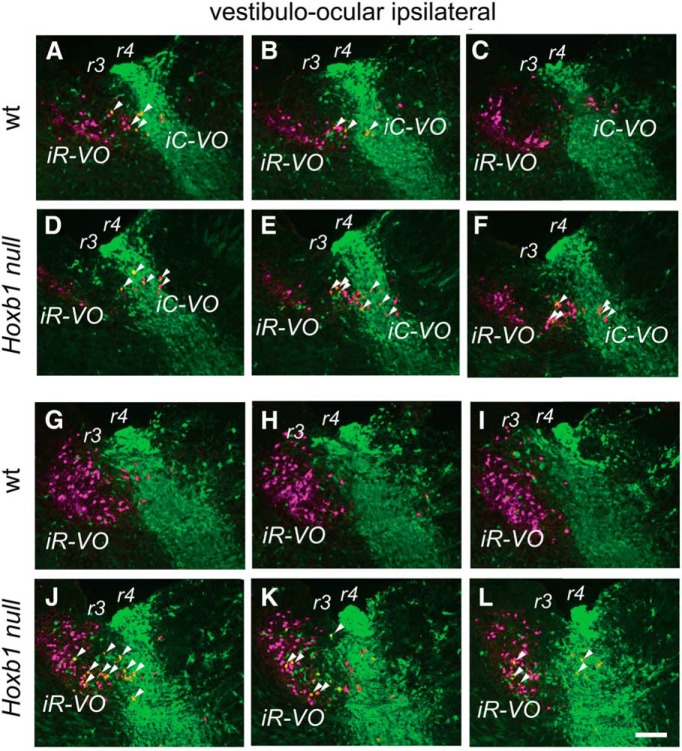
Effects of *Hoxb1*-null mutation on vestibulo-ocular neuron groups. ***A–F***, No loss of iC-VO or scattered ipsilateral VO neurons in the *Hoxb1*-null mouse. ***A–C*** are a series of parasagittal sections in a wild-type (wt) mouse embryo showing the presence of the few iC-VO neurons in r4 and scattered ipsilateral VO neurons in r3. Some of each of these can be seen to be r4-derived (examples are indicated by white arrowheads). ***D–F*** are a similar series of sections in the *Hoxb1*-null mutant, in which the situation is similar to that in the wild-type: there is no indication that iC-VO or scattered ipsilateral VO neurons are lost, and some of each are r4-derived. ***G–L***, Ectopic presence of r4-derived neurons in the region containing the iR-VO group. ***G–L*** present a series of parasagittal sections through the region containing the iR-VO group in wild-type (***G–I***) and *Hoxb1*-null (***J–L***) mice. In this region, *Hoxb1*-null mutants have ectopic r4-derived neurons (examples are indicated by white arrowheads) not seen in wild-type mice. RDA/BDA labeling is depicted by magenta, YFP immunolabeling is depicted by green, and double labeling appears as varying hues of yellow and orange. Scale bar, 200 µm.

In contrast to the essentially complete absence of r4-derived vestibulospinal neurons, the r4-derived population of reticulospinal neurons was depleted, but not absent, in both r4 and r5 of *Hoxb1*-null mice (Fig. 3*K–V*). Similarly, we still found r4-derived scattered ipsi VO neurons in r3 and iC-VO neurons in r4 of the mutant mouse (Fig. 4*A–F*, examples indicated by arrowheads). Thus, not all r4-derived vestibular projection neurons are lost when Hoxb1 function is eliminated. As expected, the cR-VO and cC-VO groups, which do not derive from r4, were not affected (data not shown).

We nevertheless found that a sizeable group of r4-derived YFP^+^ neurons had migrated into r3 in the region of the iR-VO group in the *Hoxb1*-null mutant (Figs. 3*I*,J, 4*J–L*). None of these, however, projected to the spinal cord, indicating that they had a non-vestibulospinal phenotype (Fig. 3*I*,*J*). Indeed, some of them could be retrogradely labeled from the ascending MLF, indicating a vestibulo-ocular (or potentially vestibulothalamic) phenotype (Fig. 4*J–L*). It remains to be determined whether these neurons represent an overproduction of the scattered ipsi VO and/or iR-VO neurons or LVST neurons that have been transfated to another phenotype.

### Loss of vestibular efferent neurons in *Hoxb1*-null mice

**Figure 5.**
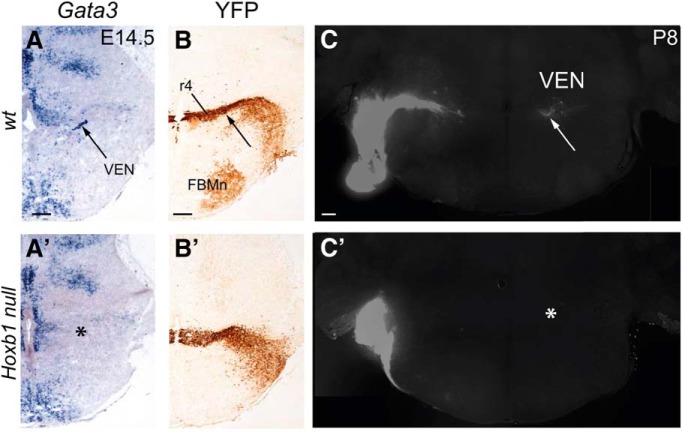
Loss of vestibular efferent neurons in the *Hoxb1*-null mutant. ***A–B'***, *In situ* hybridization for *Gata3* transcripts (***A***, ***A'***) and *Hoxb1*-driven expression of YFP (***B***, ***B'***) in wild-type (wt) and *Hoxb1*-null mouse embryos. *Gata3 in situ* hybridization in wild-type mice labels the vestibular efferent neurons (VEN) in the specific region of r4 where they differentiate (***A***, ***B***, arrows). In *Hoxb1*-null mice, this region is devoid of *Gata3* expression (***A'***, *). The application of DiI to the peripheral nerves in the inner ear at P8 retrogradely labels vestibular efferent neurons in wild-type mice (***C***, VEN), but not in *Hoxb1*-null mice (***C'***, *).

Another population of vestibular neurons is the group of cholinergic vestibular efferent neurons that innervates hair cells and calyx afferent endings in the vestibular end organs in the inner ear (Highstein 1991). Vestibular efferent neurons, similar to cochlear efferent neurons, originate from ventral r4 and express high levels of *Gata2* and *Gata3* (Tiveron et al., 2003). No *Gata3* expression was detected in the vestibular efferent neuron domain in E14.5 *Hoxb1*-null mice (*n* = 3; Fig. 5*A*,*A*'), commensurate with an absence of inner ear efferent neurons (Di Bonito et al., 2013a). To confirm the absence of vestibular efferents at postnatal stages when connectivity is established, DiI crystals were inserted into the inner ears of wild-type mice (*n* = 3) and *Hoxb1*-null mice (*n* = 3) at P8. No efferent neurons were retrogradely labeled at this stage in the mutant mice (Fig. 5*C*,*C*'), confirming their absence and excluding the presence of any other ectopic source of efferent input to the inner ear. Thus, vestibular projection neurons and vestibular efferent neurons derived from r4 are critically dependent on the expression and function of Hoxb1 protein.

### Behavioral deficits and compensation in *Hoxb1*-null mice during the early postnatal period

To investigate whether and how the deficits in the vestibular system observed in *Hoxb1*-null mice affect general motor behavior and balance, we followed a group of *Hoxb1*-null mice from early postnatal stages to adulthood.

**Figure 6.**
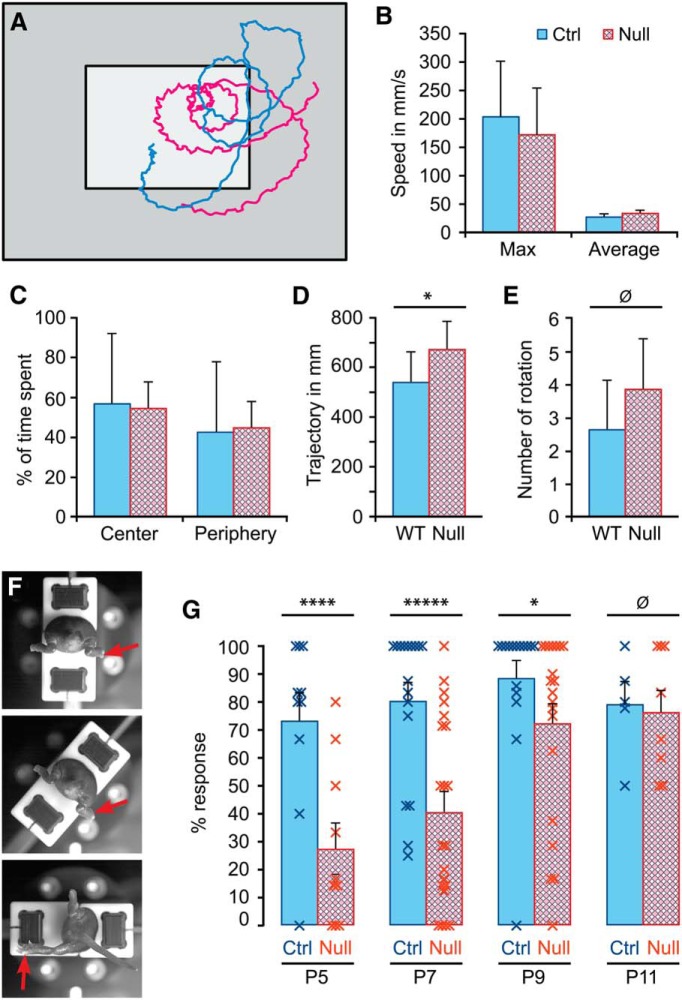
Behavioral effects of the *Hoxb1*-null mutation in young mice. ***A–E***, Open field swimming test. ***A***, Typical swimming trajectories of wild-type (blue) and *Hoxb1*-null mice (red). ***B***, Comparison of maximum and average swimming speeds. ***C***, Comparison of time spent within the central region (***A***, light gray) and peripheral region (***A***, dark gray). ***D***, Comparison of average total swimming distances (trajectory). ***E***, Comparison of average number of turns (rotations). ***F***, ***G***, Hindlimb vestibular reflex test. ***F***, An example of the test. A wild-type mouse pup mounted in the rotation device is shown from the rear. At rest (top), the pup holds its hindlimbs in a flexed position and its tail straight back. Red arrow indicates the right hindlimb. No movement is detected at the midpoint of the rotation (middle), but by full rotation (bottom) a marked extension of the right hindlimb has been elicited, and the tail has deviated to the same side and upward. ***G***, Comparison of the average response rate in the hindlimb vestibular reflex test from P5 to P11. Error bars represent SEM. *p < 0.1, ****p < 0.005, *****p < 0.001.

Testing general motor capability in early postnatal life is challenging because during the first postnatal week rodents are not strong enough to bear their own weight with extended limbs (Muir 2000), and motor function develops gradually thereafter (Geisler et al., 1993; Brocard et al 1999; Clarac et al., 2004; Lelard et al., 2006). Thus, specially designed tests need to be used. Here, we used an open field swimming test to first assess general motor capacity, and a specially designed body rotation device to test specifically the vestibulospinal reflex as it manifests in the hindlimbs (Fig. 6*A–G*).

As assessed by the open field swimming test at P5, general motor capacity was not affected in any decisive way. *Hoxb1*-null mice (*n* = 9) did not differ from control mice (*n* = 6) in average or maximum speed of swimming, in the proportion of time spent swimming in the most central part of the pool versus the periphery, or in the number of turns made during the swimming trajectory (Mann–Whitney *U* test, *U* = 16, *p* = 0.12; Fig. 6*A–C*,*E*). *Hoxb1*-null mice did, however, swim for moderately longer overall distances than control mice during the 20 s swimming session (Mann–Whitney *U* test, *U* = 12, *p* = 0.04; Fig. 6*D*). It is not clear what caused this difference, but it runs counter to any gross motor deficit.

In contrast, the vestibulospinal reflex in the hindlimbs was strongly depressed in *Hoxb1*-null mice relative to control mice when tested at P5 (*Hoxb1*-null mice, *n* = 9; wild-type control mice, *n* = 10; Mann–Whitney *U* test, *U* = 14, *p* = 0.0047) and P7 (*Hoxb1*-null mice, *n* = 21; wild-type control mice, *n* = 14; Mann–Whitney *U* test, *U* = 55, *p* = 0.0008; Fig. 6*F*,*G*). The extension of the ipsilateral leg typically elicited in control mice by a unilateral 90° rotation in the longitudinal axis was elicited only about half as often in *Hoxb1*-null mice at these stages. Strikingly, the rate recovered to control levels by P11, indicating a substantial compensation (*Hoxb1*-null mice, *n* = 8; wild-type control mice, *n* = 5; Mann–Whitney *U* test, *U* = 19, *p* = 0.27; Fig. 6*G*).

### *Hoxb1*-null mice display perturbed motor behaviors as adults

**Figure 7.**
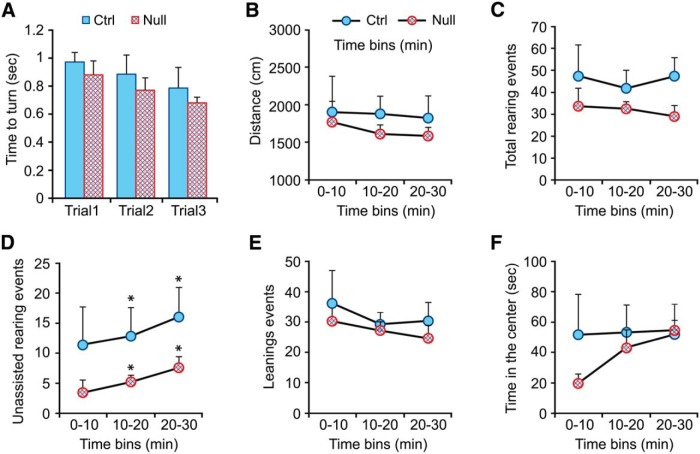
Behavioral effects of the *Hoxb1*-null mutation in adult mice: righting reflex and open field test. ***A***, Comparison of time required for the righting reflex in wild-type mice (blue) and *Hoxb1*-null (red) mice. ***B–F***, Open field behavior. Naive *Hoxb1*-null mice and their littermate controls were tested during 30 min in the open field, and total distance was scored for three consecutive 10 min time epochs for distance covered (***B***), total rearing events (***C***), unsupported rearing (***D***), leaning (rearing supported against the wall of the arena, ***E***), and time spent in the central zone of the arena (***F***). Error bars represent SEM. Significant differences with respect to control mice were calculated using the PLSD Fischer test. *p < 0.05 (second and third time bins in D).

**Figure 8.**
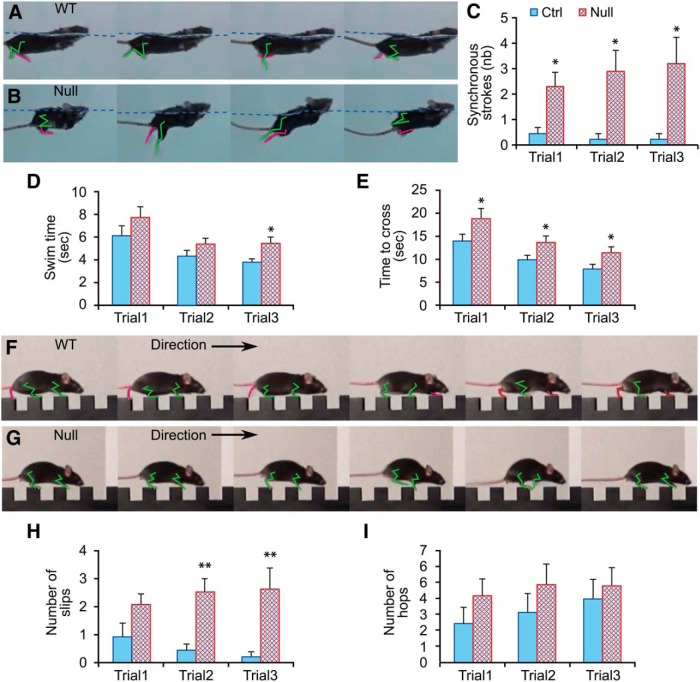
Behavioral effects of the *Hoxb1*-null mutation in adult mice: linear swimming test and notched beam test. ***A–C***, Linear swimming test. ***A***, ***B***, Examples of alternating hindlimb swimming movements in wild-type mice (***A***) and synchronous hindlimb swimming movements in *Hoxb1*-null mice (***B***). Green and red traces delineate respectively the hindlimb closest to and farthest from the camera. ***C***, Comparison of average number of synchronous hindlimb strokes. ***D***, Comparison of the average time required to swim the total distance of 1 m. ***E–I***, Notched beam test. ***E***, Comparison of average time required to traverse the beam. ***F***, ***G***, Examples of alternating and synchronous locomotor movements in wild-type and *Hoxb1*-null mice, respectively. ***H***, Comparison of the number of slips made while traversing the beam. ***I***, Comparison of the number of hops made while traversing the beam. Error bars represent SEM. Significant differences with respect to control mice were identified using the PLSD Fisher test: *p < 0.05; **p < 0.01.

To investigate whether the compensation in the vestibulospinal reflex observed by P11 was maintained in adult *Hoxb1*-null mice, we performed a series of behavioral tests, sensitive to potential deficits in balance, motor coordination, and proprioception, on a subgroup of the control and *Hoxb1*-null mice previously tested at early postnatal stages. The tests included the contact righting test, the open field test, the linear swimming test, and the notched beam test, which are all presented for *n* = 10 *Hoxb1*-null mice and *n* = 7 wild-type control mice (Figs. 7, 8).

Focusing initially on potential perturbation of the vestibular system, we noted that the righting reflex assessed by the contact righting test, known to be a sensitive measure of equilibrium, was not impaired in *Hoxb1*-null mice (Student’s *t* test, *p* > 0.4 for each trial; Fig. 7*A*). Nor did *Hoxb1*-null mice display head tilting or circling behaviors frequently associated with different types of vestibular dysfunctions (data not shown). Thus, the compensation in the vestibulospinal reflex seen during the second postnatal week evidently translated into a generally normalized capacity for vestibular function in the adult.

In addition, we found no significant difference in distance covered in the open field test over the entire test period (*Hoxb1*-null mice, 49.5 ± 5.7 m/30 min vs control mice, 50.3 ± 7.6 m/30 min; Student’s *t* test, *p* = 0.9; Fig. 7*B*), but there was a tendency for *Hoxb1*-null mice to rear on their hindlimbs less often than control mice (*Hoxb1*-null mice, 94.9 ± 12.8 rears/30 min vs control mice, 125.0 ± 24.2 rears/30 min; Student’s *t* test, *p* = 0.10; Fig. 7*C*). In a more detailed analysis, we distinguished between rearing without leaning against the wall of the arena (hereafter called “unsupported rearing”) versus rearing while leaning against the wall (hereafter called “leaning”). Unsupported rearing was significantly less frequent in *Hoxb1*-null mice relative to control mice over the entire test period (*Hoxb1*-null mice, 15.4 ± 4.2 unsupported rears/30 min vs control mice, 43.4 ± 13.4 unsupported rears/30 min; Student’s *t* test, *p* = 0.03; Fig. 7*D*), whereas there was no difference in the frequency of leaning (*Hoxb1*-null mice, 79.5 ± 10.9 leans/30 min vs control mice, 92.8 ± 15.6 leans/30 min; Student’s *t* test, *p* = 0.4 for each time bin; Fig. 7*E*). The frequency of unsupported rearing was significantly lower in each time bin during the test period (Student’s *t* test, *p* = 0.1, *p* = 0.03, and *p* = 0.02 for 0-10, 10-20, and 20-30 time bins, respectively; Fig. 7*D*), and was not correlated with reduced time spent in the central part of the arena, which did not differ significantly during the test period (Student’s *t* test, *p* > 0.2 for each time bin; Fig. 7*F*). Thus, the difference in unsupported rearing of *Hoxb1*-null mice cannot be attributed to perturbed emotional processing (e.g., higher anxiety, motivating them to stay away from the most open area of the arena and close to the walls). It is more likely due to a more subtle deficit in balance that is especially evident when the entire weight of the body is carried by the hindlimbs.

To assess motor capability with less dependence on weight-bearing equilibrium, we performed the linear 1 m swimming test, which elicits non-weight-bearing locomotion in which hindlimbs drive forward propulsion while forelimbs are held in a static flexed position (Fig. 8*A**–E*). *Hoxb1*-null mice displayed the same body position and head orientation during swimming as control mice (Fig. 8*A*,*B*), but instead of continuously using alternating hindlimb movements, they occasionally extended their hindlimbs synchronously (Fig. 8*B*; Videos 1, 2). Interposition of synchronous hindlimb movements was significantly more frequent in *Hoxb1*-null mice than in control mice (RMANOVA, *F*_(1,15)_ = 10.5, *p* < 0.005 for main effect of the *Hoxb1*-null mutation; Fig. 8*C*). Because these synchronous hindlimb movements interrupted the smooth forward motion of swimming, their generation is likely related to the tendency for *Hoxb1*-null mice to take longer to swim the 1 m distance, a time that became statistically significant on the last trial (*Hoxb1*-null mice, 3.8 ± 0.3 s vs control mice 5.4 ± 0.9 s; *p* = 0.03; Fig. 8*D*).

Also in support of a motor deficit not related only to balance per se, *Hoxb1*-null mice used significantly more time to traverse the 1 m notched beam (Fig. 8*E–G*), a difference evident in all three trials, as documented by RMANOVA (*F*_(1,15)_ = 6.0, *p* = 0.03 for main effect of *Hoxb1*-null mutation) and *post hoc* analyses. These longer times were associated with an increased number of slips during stepping across each notch (RMANOVA, *F*_(1,15)_ = 11.2, *p* = 0.004 for the main effect of the *Hoxb1*-null mutation; Fig. 8*H*). We also assessed hindlimb coordination during the traverse of the notched beam, and found that although *Hoxb1*-null mice tended more often than wild-type control mice to display hopping when moving from one step of the beam to the next, with synchronous extensions of both hindlimbs analogous to the synchronous strokes exhibited during swimming (Fig. 8*G*,*I*; Videos 3, 4), this difference did not reach significance (*F*_(1,15)_ = 1.1, *p* = 0.3 for main effect of the *Hoxb1*-null mutation).

Thus, despite the substantial early compensation in vestibular function, *Hoxb1*-null mice exhibit abnormalities in proprioceptive and locomotor behavior as adults, which are likely to be due to either the loss of the LVST, the deficit in reticulospinal projections, or both.

## Discussion

### General summary

Using a combination of retrograde labeling and genetic fate mapping, we here provide an extensive characterization of the contribution of r4 to the vestibular system, and in particular to specific populations of vestibular projection neurons. We also demonstrate a contribution of r4 to the reticulospinal system, which, though minor, is important when considering the physiological and behavioral effects of perturbing r4 development. We demonstrate that inactivating *Hoxb1*, a master gene involved in imparting r4 identity (Studer et al., 1996, Di Bonito et al., 2013a,b), leads to the absence of several r4 derivatives (including vestibular efferent neurons and specific subpopulations of vestibulospinal neurons), and partially affects other r4 derivatives (including reticulospinal and vestibulo-ocular neurons). We also make the first assessment of the behavioral effects that result from this genetic perturbation of r4.

### Segmental pattering of vestibular projection neurons: the role of r4 and *Hoxb1*

Where earlier studies have only been able to presume the relationship between neuron groups and the r4 boundaries due to technical limitations in fate mapping, we have been able to define explicitly which neurons derive from r4 and which do not. Thus, we can now say with certainty that the vast majority of, and possibly all, LVST neurons derive from r4, as does a specific subpopulation of cMVST neurons. We also have strong evidence that some iC-VO neurons originate in r4, as do the scattered ipsi VO neurons in r3. Interestingly, our study has identified a population of reticulospinal neurons that derives from r4, some of which migrate into r5. The functional role of this particular reticulospinal neuron subpopulation remains to be defined. Finally, r4 also gives rise to the vestibular efferent neurons, but these represent a quite different category of neurons from the vestibular projection neurons, as they are more closely related to motoneurons. They originate from a more ventral domain, extend their axons to the periphery, use acetylcholine as the principal neurotransmitter, and modulate the contractile state of their target hair cells.

**Figure 9.**
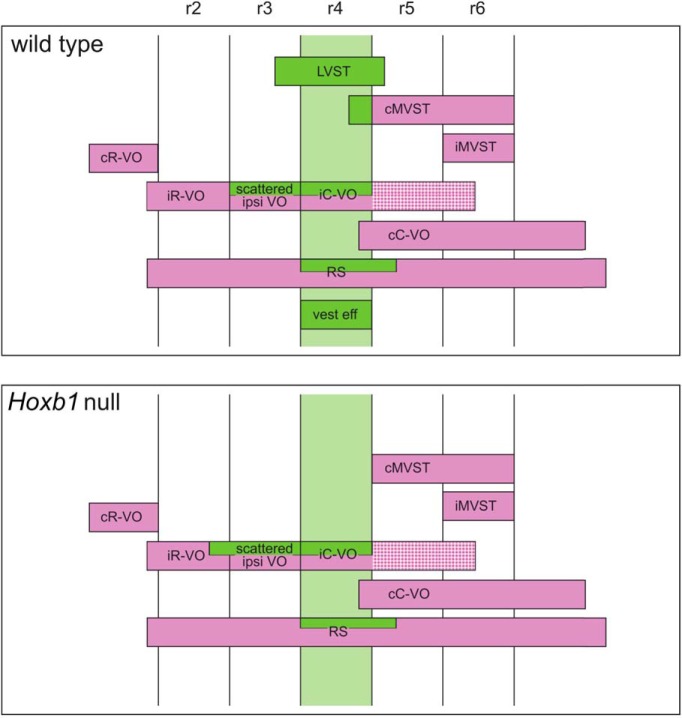
Summary of segmental origins of vestibular projection neurons, vestibular efferent neurons, and reticulospinal neurons in the wild-type mice (top), and of the phenotype of the same neuron populations in the *Hoxb1*-null mutant mice (bottom).

This places the contribution of r4 to the patterning of vestibular projection neurons into relief. Rhombomere 4 lies about midway along the rostrocaudal extent of the vestibular nuclear complex and represents in several respects a key domain in patterning vestibular projection neurons. It gives rise to the principal vestibulospinal neuron group, the LVST, but to few vestibulo-ocular projection neurons. The other vestibulospinal neuron groups, coursing in the MVST, derive predominantly from the more caudal r5 and r6, although a portion of the cMVST group derives from r4. Thus, within the context of vestibular projection neurons, r4 is primarily related to vestibulospinal specification, whereas vestibulo-ocular projection neurons derive predominantly from more rostral and more caudal rhombomeres (Fig. 9).

The lack of LVST neurons and r4-derived cMVST neurons in the *Hoxb1*-null mutant (Fig. 9) suggests either an outright failure of their generation from progenitor cells, their subsequent early death, or their differentiation to another, non-vestibulospinal phenotype. Because the loss of Hoxb1 function prevents the maintenance of r4 identity (Studer et al., 1996; Gavalas et al., 1998; Weicksel et al., 2014) and mainly transfates r4 into r3 (Di Bonito et al., 2013a,b), the differentiation of r4-derived neurons into r3-related fates would be expected. As noted, a small number of non-r4-derived spinally projecting neurons was present in the LVST domain in the *Hoxb1*-null mutant. Their origin and function remain to be determined, but they could represent some iMVST neurons that have attained abnormally rostral positions.

On the other hand, vestibulo-ocular neurons and a minor population of reticulospinal neurons derived from r4, are not wholly eliminated in the *Hoxb1*-null mutant. These findings prompt several hypotheses, as follows: (1) vestibulo-ocular and reticulospinal neurons are less dependent than vestibulospinal neurons on *Hoxb1* expression; (2) differentiation and maintenance of vestibular projection neurons is characterized by a hierarchy wherein vestibulo-ocular neurons represent a default phenotype that is overridden by Hoxb1 function to create a vestibulospinal phenotype; and (3) r3-like vestibulo-ocular and reticulospinal neurons are generated in r4 in the absence of *Hoxb1*, as is the case with other r4-derived neuronal populations such as facial branchial motoneurons, auditory neurons, and trigeminal neurons (Studer et al., 1996; Di Bonito et al., 2013a,b). This last hypothesis would also explain why LVST and cMVST neurons, which normally originate from r4 but not r3, fail to form in *Hoxb1*-null mice (the mutant r4 becomes r3, which does not give rise to vestibulospinal neurons), whereas vestibulo-ocular and reticulospinal neurons, which normally originate from both r3 and r4, are still generated from r4 in the *Hoxb1*-null mutant (the mutant r4 becomes r3, which does give rise to these neuron types). To ultimately demonstrate transfating, we would need rhombomere- and group-specific markers of the vestibular projection neurons, which are not yet available.

### The LVST group as a developmental and functional entity

Earlier studies have highlighted the fact that the LVST neuron group and the lateral vestibular nucleus (LVN) are not equivalent, as many vestibulospinal neurons projecting in the MVST are located in the ventral LVN (for review, see Akaike, 1983). The noncongruence of the LVST neuron group and the LVN has been demonstrated explicitly in the chicken embryo by direct comparison of hodological and cytoarchitectonic domains, which has shown that parts of the cMVST, iR-VO, and cC-VO neuron groups (and potentially other vestibular projection neuron groups) also lie within the LVN (Díaz et al., 2003). More recently, this lack of congruence has been noted in tracing studies of the LVST in the adult mouse (Liang et al., 2014). Although a common practice in the literature, we strongly recommend that in the future the source of the LVST be denoted not as the LVN, but rather as the LVST neuron group, which is correct and precise.

That the LVST group derives essentially in its entirety from r4 implicates *Hoxb1* as a core molecular component in the genetic program for LVST specification. Nevertheless, some LVST neurons migrate from r4 into r3 and r5, raising the possibility that these subpopulations differ functionally from the main subpopulation that remains in r4 (Fig. 9). This could be related to the topographic organization of the LVST and its spinal targets, in which the most rostral and caudal LVST neurons connect respectively to cervical and lumbar spinal levels (Shamboul, 1980). Other studies have shown that the LVST neurons exhibit further heterogeneity in their size, clustering, and dendritic arbors (Glover and Petursdottir, 1988; Liang et al., 2014), indicating that their r4-specific origin does not impart a monolithic pattern of differentiation.

Of the three vestibulospinal neuron groups, only the LVST group projects beyond cervical and upper thoracic segments (Wilson and Peterson, 1978; Kasumacic et al., 2010), and activity in the LVST is known to activate primarily extensor muscles. Thus, it is thought that the vestibulospinal reflex in the hindlimbs is elicited predominantly by impulses traveling in LVST axons that synapse on extensor motoneurons, or interneurons immediately presynaptic to these, in the lumbar spinal cord. Numerous studies have investigated the behavioral effects of peripheral vestibular lesions, but, to our knowledge, this is the first time the central source of the LVST has been eliminated bilaterally, certainly using nonsurgical techniques. Using a dedicated vestibulospinal reflex test device for young mouse pups, we show that the bilateral lack of the LVST greatly diminishes the fidelity of the vestibulospinal reflex in the hindlimbs.

Using non-weight-bearing swimming to test general motor capability in the *Hoxb1*-null mice at the same age when the vestibulospinal reflex is profoundly affected (P5) revealed no obvious differences from controls in a number of variables, including maximum speed, average speed, and number of turns. Moreover, the total trajectory traversed was ∼20% higher in the *Hoxb1*-null mice. Thus, the bilateral loss of the LVST had little effect on general motor capability within the limits of the behavioral tests that we used, suggesting that the LVST is primarily concerned with weight-bearing motor functions and particularly with the limb extension that typifies the vestibulospinal reflex.

### Recovery of the vestibulospinal reflex

Though greatly diminished at P5, the vestibulospinal reflex in the hindlimbs gradually recovered over the ensuing week. Several possible explanations exist, including the growth of the iMVST or cMVST beyond their normal longitudinal span, a vestibulospinal function for the few non-r4-derived spinally projecting neurons seen in the LVST domain, or a greater recruitment of vestibulo-reticulospinal connections into the reflex pathway, an explanation that we consider to be the most probable. Though intrinsically slower than the direct vestibulospinal pathway, connections from vestibular nuclei (including the ventral part of the LVN, where many LVST neurons reside) to the medial reticular formation are thought to provide a parallel pathway to the spinal cord (Peterson and Abzug, 1975). Because reticulospinal projections are also well developed by birth in the mouse (Leong et al., 1984; Auclair et al., 1999), they could clearly provide an existing substrate for transmitting vestibular signals to the lumbar cord even in the absence of the LVST.

### Motor deficits in the adult—possible additional roles for the LVST in regulating motor function

Despite profound compensation of the vestibulospinal reflex during early postnatal life, other functional deficits in the motor system were evident in the adult. Most of these did not appear to affect balance or equilibrium per se, but rather the coordination of limb movement that is required to perform more demanding motor tasks. Adult *Hoxb1*-null mice had a normal righting reflex and did not exhibit abnormal circling behavior, suggesting that the early compensation seen for the vestibulospinal reflex was persistent, bilateral, and effective in a more general vestibular context. They did, however, show a decrease in unsupported rearing in the open field, suggesting that the compensation was not sufficient to provide effective balance in all situations.

Nevertheless, the most obvious behavioral deficits were less balance related. For example, during swimming, adult *Hoxb1*-null mice were more likely to use synchronous hindlimb kicks than control mice. There was a tendency for synchronous activation of the hindlimbs and the forelimbs to be more prevalent in *Hoxb1*-null mice when navigating the notched beam as well. This interesting phenotype suggests a link to the spinal locomotor network, which typically generates alternating hindlimb movements in adults (Kiehn, 2006), but synchronous hindlimb movements during fetal life (Nakayama et al., 2002), during situations in which the balance between excitation and inhibition between the left and right sides is altered (Restrepo et al., 2011) or when specific elements of the locomotor central pattern generator are perturbed genetically (Lanuza et al., 2004; Crone et al., 2008; Zhang et al., 2008; Talpalar et al., 2013; Shevtsova et al., 2015). The other deficit that was particularly evident was the increased number of slips made by *Hoxb1*-null mice on the notched beam, which most likely contributed to the increased time required to traverse the beam. This phenotype could reflect a variety of potential deficits, including in the dynamic coordination of hindlimb muscles imposed by the spinal locomotor network, but also in the various mechanoreceptive and proprioceptive inputs that mold locomotor output according to the demands of the substrate.

Although both the swimming and the notched beam tests were originally conceived to evaluate limb coordination, they could also be affected by perturbed balance and equilibrium, or by proprioceptive deficits, as has been described previously for the notched beam test (Shelton et al., 2008). Moreover, we have shown that another descending input, from the small number of r4-derived reticulospinal neurons, is also depleted in the *Hoxb1*-null mice. Thus, we cannot rule out that the non-balance-related motor deficits result from perturbation of this pathway instead of from the loss of the LVST. Nevertheless, in a recent report using trans-synaptic rabies virus tracing, Bourane et al. (2015) provide evidence for synaptic connections between the vestibular nuclei and a set of dorsal horn interneurons involved in light touch and fine motor control. Thus, we propose that the non-balance-related motor deficits in adult *Hoxb1*-null mice might reflect the following two additional roles for the LVST: (1) providing a descending channel for introducing bias in the activation of the two hindlimbs by the spinal locomotor network, thus contributing to alternation as opposed to coactivation; and (2) regulating local sensorimotor circuits in the spinal cord according to the dynamic balance of extensor and flexor muscle activity required to move the hindlimbs in precisely coordinated patterns. Thus, instead of operating solely within the realm of balance and equilibrium, the LVST may provide a rapid channel for fine-tuning extensor muscle activity in a more global context.

Video 1. Swimming exhibited by a wild-type mouse. 10.1523/ENEURO.0096-15.2015.video.1

Video 2. Swimming exhibited by a *Hoxb1*-null mutant mouse. 10.1523/ENEURO.0096-15.2015.video.2

Video 3. Traversal of the notched beam by a wild-type mouse. 10.1523/ENEURO.0096-15.2015.video.3

Video 4. Traversal of the notched beam by a *Hoxb1*-null mutant mouse. 10.1523/ENEURO.0096-15.2015.video.4

## Synthesis

The decision was a result of the Reviewing Editor Muriel Thoby-Brisson and the peer reviewers coming together and discussing their recommendations until a consensus was reached. A fact-based synthesis statement explaining their decision and outlining what is needed to prepare a revision is listed below. The following reviewers agreed to reveal their identity: Mathieu Beraneck, Gary Gaufo

Summary

Dear authors,

The two reviewers and myself agree on considering your paper potentially suitable for publication in eNeuro after proper revisions are made. We recognize the high quality of the work, the advances it will provide in the field and beyond. We mainly recommend to:

- be more rigorous on the details of the animals populations used for different tests,

- at least cite the Goulding/Jessell work in the discussion,

- carefully update the reference list (several citations are missing).

Also we do think that the movies are indisputably nicely illustrating the data and are useful for the readers.

Below you will find the detailed comments of the two reviewers.

Sincerely yours,

Muriel Thoby-Brisson

Reviewer 1:

This paper investigates the role of the Hoxb1 gene in the developmental pattern of vestibular neurons. The authors first identify several groups of neurons which originate totally or partially on the rhombomere 4 (r4). The experimental approach then takes advantage on the use of transgenic mouse on in particular on Hoxb1 null strain to determine which subpopulations of neurons depend on it. They demonstrate that neurons from the lateral vestibulospinal tract, contralateral medial vestibulospinal tract, and efferent neurons depend particularly on r4. In addition, the authors investigate the behavioral deficits during early postnatal period and adulthood. Results show that HoxB1 null mice exhibit strong deficits during early development at P5, which compensate during the following days. In adults, balance appears mostly unaffected while more general motor behavior shows impairments. The authors conclude on the role of Hoxb1 in patterning the vestibular system and on the LVST, which would have a particular importance for regulation of locomotion and interlimb coordination.

The authors elegantly take advantage of mouse genetics to investigate the subpopulations of vestibular neurons which constitute vestibular descending pathways. The manuscript is well written, comprehensive; the conclusions made from the data are fair and prudent and largely supported by the data, the figures and videos are well designed. My main concern is on the heterogeneity of the group of mice which mix male/female; heterozygous/homozygous, and 13/28 weeks old. Although I believe, as do the authors, that this heterogeneity does not affect the conclusion of the paper, it makes the behavioral data too sloppy. I encourage the author to raise the rigour of behavioural data set so that it matches the overall very good quality of the work, both from a technical and scientific point of view.

Major comments:

1-Please state the total number of animals used in the study, and the number of animals used for each data set.

2-For behavioral tests, do not merge Hoxb1 +/- with homozygous animals. If you need to ensure sufficient statistical power, please add more Hoxb1+/+ animals.

3-Most testing were performed at age 13-16 weeks, except for 4 aged of 28 weeks. I recommend keeping the age constant.

Minor comments:

Abstract: last sentence is weak, what do the authors mean by "more complex"?

Significance statement: again, last sentence needs to be rewritten to specify what the authors mean by "additional roles"?

Introduction:

Page 3, 1st paragraph: Remove the last sentence "the contribution....mortality".

Page3, 2nd paragraph: First sentence, add "vestibular descending outputs"

Page 4, 1st paragraph: delete "and actually operates in health and disease".

Page 4, 2nd paragraph: please add a citation after "vestibular and bulbospinal projection neuron groups".

Page 4, 3rd paragraph: the 11 lines text "we provide....is compromised" is too long and reads like an abstract. Please edit.

Methods:

Page 5: references are missing: Srivinas et al. 2001; Di Bonito et al.a, b 2013...please carefully check bibliography.

Page 5, 3rd paragraph: the European regulation is now directive 2010/63/EU. Please correct.

Page 6, 4th paragraph: could you detail your approach to insert the crystal into the inner ear? What is the rational for incubating the head 1 to 3 months? Methods should help other group to reproduce the data if needed. Would you recommend 1 or 3 month incubation? Please edit accordingly.

Page 8, 2nd paragraph: please state the speed and angle of rotation. Please also state the analysis criteria.

Results:

Page 11, 1st paragraph: remove "definitive".

Page 16, 2nd paragraph: suggest removing mentions of head tilting and circling, as the link with VSR is not evident.

Page 20, 2nd paragraph: are all efferent neurons using acetylcholine? Please also specify the "modulation of the mechanical properties of their target cells".

Figure 1: suggest showing LVST on panel G, and maybe RS and LVST boundaries?

Reviewer 2:

The developing hindbrain is a tractable model to study how the evolutionarily conserved Hox genes pattern the various neuronal systems along the anterior-posterior axis of the neural tube. To date, the majority of studies have focused on defining the molecular and cellular mechanisms that contribute to the Hox-dependent neuronal patterning programs. Few studies have embarked on the physiological or behavioral consequences of Hox gene mutation(s). The present manuscript addresses this important issue, focusing on the contribution of Hoxb1 on the development and function of vestibular system.

Building on previous studies in mouse and chick embryos, the current study establishes the rhombomeric (r) origin of the various vestibular neuronal groups (nuclei). This is done with greater precision and exquisite detail using an r4-specific Cre/Rosa-reporter system and retrograde labeling. Their principal finding is that neurons that make up the lateral vestibulospinal tract (LVST) derives solely from r4. Furthermore, they confirm that the specification of the LVST is dependent on the r4 determinant, Hoxb1.

Having convincingly established the origin and Hoxb1-dependence of the LVST, the study carefully dissected the behavioral consequence of this phenomenon in postnatal and adult mice. Surprisingly, Hoxb1-mutant mice showed normal gross motor behavior (weight bearing equilibrium) that was hypothesized to be associated or dependent on the LVST. Instead, they uncovered using a battery of behavioral assays that the LVST contributes to a specific aspect of locomotion, alternating and/or right-left limb movements. Although the regulation of the non-weight bearing locomotion cannot be solely attributed to the LVST (as they stated in the manuscript), the work provides strong evidence for the contribution of the LVST. Although outside the scope of this study, ablation of Hoxb1 in the dorsal neural tube while maintaining the function of Hoxb1 in the ventral tube may dissect the contribution of the LVST from the more ventral reticulospinal tract.

Overall, the enthusiasm and recommendation for the manuscript are very high, but with a minor revision/addition. Since the most dramatic behavioral phenotype was a defect in the coordination of locomotion (right-left, asynchronous vs synchronous), the papers related to this behavior by the Goulding and Jessell groups should be cited (see below). The findings from these papers are central to the hypothesis proposed in discussion - specifically, the possibility that the LVST regulates the spinal central pattern generator(s) that are essential for coordinated locomotion.

Genetic identification of spinal interneurons that coordinate left-right locomotor activity necessary for walking movements.

Lanuza GM, Gosgnach S, Pierani A, Jessell TM, Goulding M.

Neuron. 2004 May 13;42(3):375-86.

V1 spinal neurons regulate the speed of vertebrate locomotor outputs.

Gosgnach S1, Lanuza GM, Butt SJ, Saueressig H, Zhang Y, Velasquez T, Riethmacher D, Callaway EM, Kiehn O, Goulding M.

Nature. 2006 Mar 9;440(7081):215-9.

V3 spinal neurons establish a robust and balanced locomotor rhythm during walking.

Zhang Y, Narayan S, Geiman E, Lanuza GM, Velasquez T, Shanks B, Akay T, Dyck J, Pearson K, Gosgnach S, Fan CM, Goulding M.

Neuron. 2008 Oct 9;60(1):84-96.

V1 and v2b interneurons secure the alternating flexor-extensor motor activity mice require for limbed locomotion.

Zhang J, Lanuza GM, Britz O, Wang Z, Siembab VC, Zhang Y, Velasquez T, Alvarez FJ, Frank E, Goulding M.

Neuron. 2014 Apr 2;82(1):138-50. doi: 10.1016/j.neuron.2014.02.013.
